# A bacterial ally for nitrogen-fixing biofilm: enhancing the rhizosphere colonization of *Stutzerimonas stutzeri* A1501 with surfactin-producing *Bacillus velezensis* BRI3

**DOI:** 10.1128/aem.00498-26

**Published:** 2026-05-29

**Authors:** Yaoyao Liu, Min Zhu, Guohua Zou, Changyan Yin, Yuhua Zhan, Wei Lu, Min Lin, Xiubin Ke, Yongliang Yan

**Affiliations:** 1 Biotechnology Research Institute/National Key Laboratory of Agricultural Microbiology, Chinese Academy of Agricultural Sciences 12661, Beijing, China; 2 Biotechnology Research Institute/Key Laboratory of Agricultural Microbiome (MARA), Chinese Academy of Agricultural Sciences 12661, Beijing, China; 3 Department of Agriculture and Food Science, Shijiazhuang University 71181, Shijiazhuang, China; 4 College of Agriculture, Henan University 12411, Kaifeng, China; The University of Arizona 8041, Tucson, Arizona, USA

**Keywords:** nitrogen fixation, biofilm, rhizosphere colonization, surfactin, *Stutzerimonas stutzeri*, *Bacillus velezensis*

## Abstract

Nitrogen acquisition is pivotal for plant growth. In soil ecosystems, bacterial interactions promote nitrogen assimilation and rhizosphere colonization. However, the mechanisms underlying the interactions between nitrogen-fixing microorganisms and their neighboring organisms in the environment remain unclear. Here, we demonstrate that *Bacillus velezensis* BRI3, a rhizosphere-derived strain, forms microbial synergy with *Stutzerimonas stutzeri* A1501, functioning as a facilitator. This microbial synergy greatly increases the nitrogen-fixation by 3.2-fold and rhizosphere colonization capabilities by 2.3-fold of A1501, collectively promoting plant growth in the rhizosphere. In this study, for the first time, we propose that surfactin produced by BRI3 regulates interactions among this bacterial consortium by stimulating A1501 biofilm formation. This discovery enhances the understanding of metabolic interactions between nitrogen-fixing bacteria and their neighboring organisms. Overall, we propose a strain interaction paradigm that offers a novel framework for improving nitrogen utilization and crop yield.

**IMPORTANCE:**

Nitrogen is essential for crop productivity because it directly participates in the construction of proteins and nucleic acids. Associative diazotrophs convert N₂ into NH₄^+^, yet require root biofilms and stable colonization. Owing to the complexity of the rhizospheric microbiota, a systematic understanding of microbe–microbe interactions and their impact on nitrogen-fixation capacity is still lacking. This study uncovers a novel intergeneric synergism in which *B. velezensis* BRI3 secretes surfactin that triggers *S. stutzeri* A1501 biofilm formation and concurrently elevates *nif* gene expression, thereby facilitating the integration of microbe–microbe interaction, biofilm development, and nitrogen-fixation efficiency into a single linear pathway. This phenomenon also provides a portable molecular-to-phenotypic blueprint for designing composite inoculants. Second, field trials revealed that coinoculation of these strains boosted maize growth, allowing partial synthetic-N replacement without transgenes or high costs, merely via rational strain formulation. This study highlights a transition from focusing on the ecological features of associative bacteria toward the development of deployable technology, offering a theory and prototype for sustainable agriculture.

## INTRODUCTION

Nitrogen, as an essential element for plant growth and development, is not only fundamental to the formation of key biological macromolecules such as proteins and nucleic acids but also directly governs crop yield (1). Diazotrophic microorganisms around the plant rhizosphere convert atmospheric nitrogen (N₂) into plant-available NH_4_^+^ via symbiotic, associative, and free-living nitrogen fixation pathways (2). Although symbiotic nitrogen fixation by rhizobia in legumes supplies substantial nitrogen to crops, accumulating evidence indicates that the degree of associative nitrogen fixation in Poaceae species is comparable in magnitude (3–5). Global data syntheses reveal that Poaceae species fix approximately 15.58 Tg N annually, a value comparable to the 17.91 Tg N fixed by legumes, thereby highlighting the nonnegligible agronomic contribution of associative nitrogen fixation (6, 7). Associative nitrogen fixation involves a loose mutualism between rhizospheric diazotrophs and host plants and is morphologically and physiologically distinct from legume nodule symbiosis (8). Consequently, associative nitrogen fixation has an exceptionally broad plant host spectrum and strong environmental adaptability. Research on and deployment of associative diazotrophs can provide a viable strategy for decreasing agricultural dependence on synthetic nitrogen fertilizers, thereby safeguarding food security and promoting sustainable cropping systems.

The stable rhizosphere colonization and functional expression of plant growth-promoting rhizobacteria (PGPR), including diazotrophs, are contingent upon their ability to form biofilms (9–11). Biofilms are community structures formed by microorganisms in response to environmental changes and serve as the foundation for the rhizosphere colonization of plant growth-promoting bacteria. Comprising cells and the extracellular polymeric substances (EPSs) secreted by them, biofilms can coordinate functions such as nitrogen fixation and plant growth promotion through quorum sensing (12, 13). Edwards et al. first reported a significant positive correlation between biofilm biosynthesis genes (*psl*, *pel*, and *alg*) and nitrogen-fixation genes (*nif*) within root-associated diazotrophic communities, providing empirical evidence that biofilm formation enhances the efficiency of nitrogen fixation (14). A recent polyvinylidene fluoride (PVDF) membrane-based *in situ* biofilm entrapment system demonstrated that diazotrophic biofilms formed on the membrane surface significantly increase *nifH* transcript abundance, offering a simple and reproducible approach for the direct assessment of biofilm–nitrogen fixation relationships (15). *Stutzerimonas stutzeri* A1501, a representative associative nitrogen-fixing bacterium exhibiting plant-beneficial properties and isolated from the rice rhizosphere, was employed as the main experimental strain in this study (16, 17). This species was originally described as *Pseudomonas stutzeri* but has been formally reclassified from the genus *Pseudomonas* into the new genus *Stutzerimonas* and consequently renamed *Stutzerimonas stutzeri* in a 2022 taxonomic study (18). The Gac/Rsm system and the cyclic-di-GMP (c-di-GMP) signaling pathway represent core mechanisms mediating biofilm formation in *Stutzerimonas* species (19, 20). Our previous study revealed that under nitrogen-limiting conditions, *S. stutzeri* A1501 promotes biofilm formation by upregulating genes such as *gacA* in the Gac/Rsm system and *sadc* in the c-di-GMP signaling pathway, thereby increasing rhizosphere fitness via improved surface colonization and exopolysaccharide secretion, and ultimately significantly increasing nitrogen fixation efficiency (21, 22). Moreover, A1501-mediated biofilm attachment to maize roots increases nitrogen uptake by 15%–20% (23). These findings collectively reveal a strong link between biofilm formation and nitrogen-fixation capacity in diazotrophs, and understanding this relationship is critical for improving crop productivity.

Emerging evidence shows that microbial synergy among PGPR enhances plant-beneficial functions, notably nitrogen fixation and biofilm formation (24). Zveushe et al. reported that the coinoculation of *Saccharomyces* spp. with *Bradyrhizobium japonicum* promoted soybean growth by increasing nitrogen fixation efficiency, as evidenced by increased plant height and improved root development (25). A study elucidated that microbial synergy with co-occurring soil bacteria can increase the nitrogen fixation efficiency of rhizobia through the synthesis of riboflavin (26). As a representative PGPR, the microbial synergy between *Pseudomonas* spp. and *Bacillus* spp. has been exploited for plant growth promotion and stress resistance (27). Representative research has indicated that through the production of specific metabolites, *Bacillus velezensis* SQR9 stimulates the activity of *Pseudomonas* spp. in the rhizosphere, thereby facilitating biofilm formation (28). Another report revealed that pyoluteorin-deficient *Pseudomonas protegens* Pf-5 exhibited enhanced cooperation with *Bacillus velezensis* DMW1 in various aspects, including biofilm formation, metabolite production, root colonization, control of tomato bacterial wilt disease, and synergistic interactions with beneficial bacteria in the tomato rhizosphere (29). Recent studies have revealed that surfactin synthesized by *Bacillus* spp. can act as a signaling molecule to induce the formation of bacterial biofilms, and this active substance is also an important factor promoting bacterial colonization (30–33). However, whether the contribution of *Bacillus* spp. to the biofilm formation of *Stutzerimonas* spp. is related to the surfactin synthesized by them remains unknown. *Bacillus velezensis* BRI3 is a rhizosphere growth-promoting bacterium with biocontrol activity reported in our previous study, and a variety of secondary metabolite biosynthetic gene clusters, including that for surfactin, were identified in its genome (34). Although cooperative interactions between *Bacillus* spp. and *Stutzerimonas* spp. in the plant rhizosphere have been documented, and interspecific cross-talk and coinoculation strategies have been widely exploited to enhance nitrogen fixation by diazotrophs, studies focusing on associative nitrogen-fixing bacteria such as *S. stutzeri* A1501 under nitrogen-limited conditions remain scarce. In particular, the relationships among bacterial interactions, *S. stutzeri* A1501 biofilm formation, and enhanced nitrogen fixation remain poorly understood. Here, we demonstrate that the microbial synergy between *B. velezensis* BRI3 and *S. stutzeri* A1501 accelerates nitrogen-fixing biofilm formation in *S. stutzeri* A1501. For the first time, we propose that the surfactin synthesized by *B. velezensis* BRI3 plays a crucial role in driving this process. This microbial synergy promotes maize growth under field conditions, laying the groundwork for combined-strain inoculants and sustainable agriculture.

## RESULTS

### Microbial synergy with *B. velezensis* BRI3 enhanced the nitrogen-fixation and biofilm formation capabilities of *S. stutzeri* A1501

**Fig 1:**
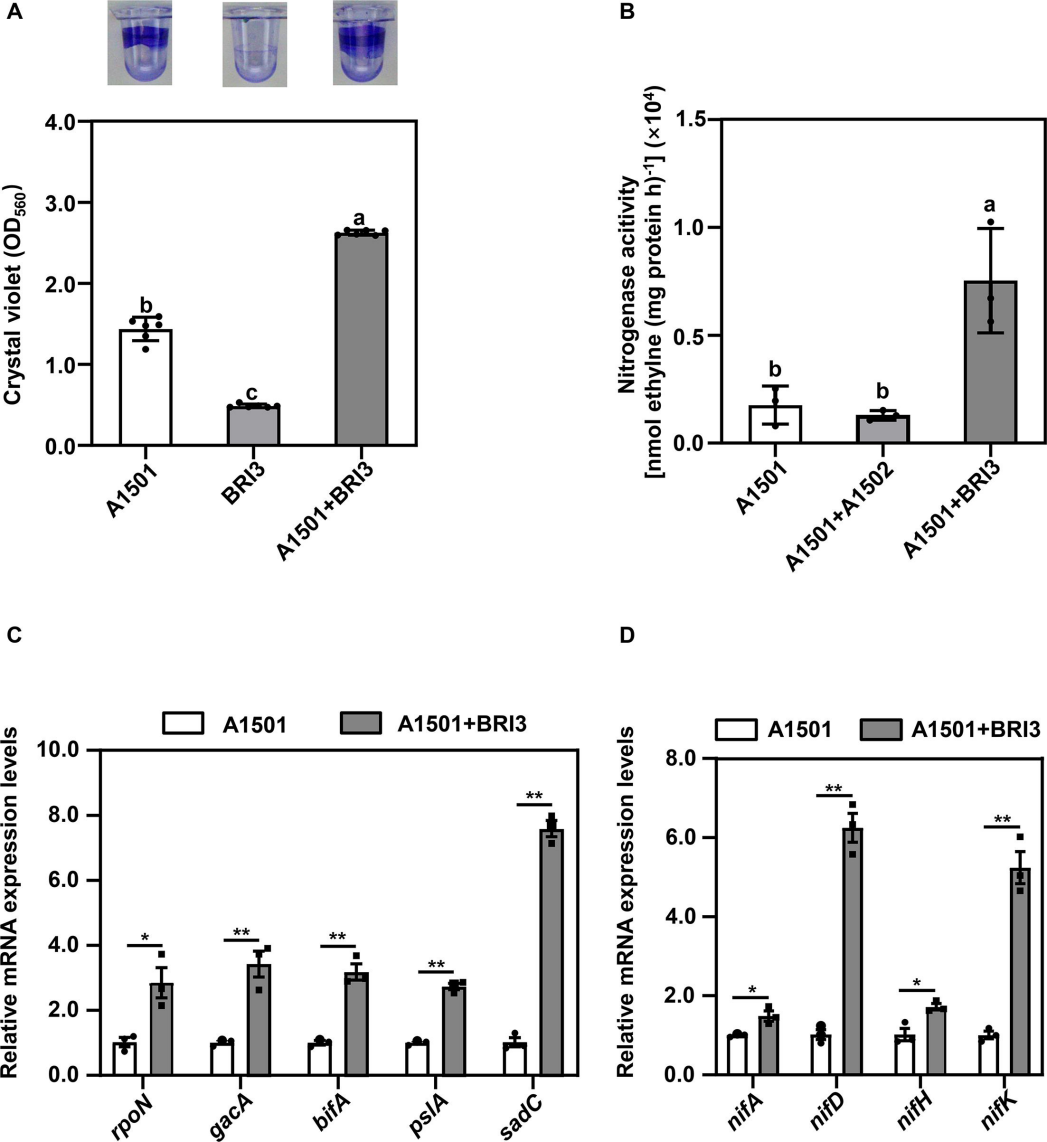
Microbial synergy enhances the nitrogen-fixing biofilm formation and nitrogen-fixing ability of *S. stutzeri* A1501 (**A**) Effect of monoculture or interaction system with *S. stutzeri* A1501 and *B. velezensis* BRI3 on the mature biofilm biomass was determined via the crystal violet (CV) method after 48 h of incubation. (**B**) Effect of monoculture or interaction system with *S. stutzeri* A1501 and *B. velezensis* BRI3 on nitrogenase activity under biofilm growth conditions. (**C**) Relative mRNA expression levels of genes related to biofilm formation in nitrogen-fixing biofilm cells under monoculture or interaction conditions. (**D**) Relative mRNA expression levels of nitrogen fixation-related genes in nitrogen-fixing biofilm cells under monoculture or interaction conditions. The data are presented as the mean ± s.d. (*n* = 3–6). Significance analysis in panels** A–B** was performed using two-way ANOVA followed by Tukey’s *post hoc* test via GraphPad Prism 8. Different letters indicate statistically significant differences (*P* ≤ 0.05). Significance analysis in panels **C–D** was performed using a *t* test. * indicates a significant difference (*P* ≤ 0.05); ** indicates a highly significant difference (*P* ≤ 0.01).

To investigate the effects of *B. velezensis* BRI3 on *S. stutzeri* A1501 under interaction conditions, the ability of different cultivation systems to form mature biofilms and fix nitrogen was evaluated 48 h post-inoculation using nitrogen-free minimal medium K (Fig. 1). The experimental results demonstrated that under nitrogen-deficient conditions, compared with A1501 monoculture, A1501 combined with BRI3 in the interaction system resulted in greater biofilm formation. Under nitrogen-deficient conditions, *B. velezensis* BRI3, which lacks an autonomous nitrogen-fixing ability, forms negligible biofilms (Fig. 1A). This result indicates that the addition of BRI3 enhances the biofilm formation ability of A1501. Notably, the level of nitrogenase activity in the biofilm system with A1501 and BRI3 interaction was 3.2-fold greater than that in the A1501 monoculture system (Fig. 1B). As a control, the nitrogen fixation-deficient strain *S. stutzeri* A1502 *nifH* mutant was combined with A1501 in the interaction system, and the nitrogenase activity in this combined system did not significantly differ from that in the A1501 monoculture. These findings indicate that the enhancement of nitrogenase activity in A1501 by BRI3 is a specific stimulatory effect. To exclude the possibility that the observed increases in biofilm formation and nitrogenase activity were caused by changes in bacterial cell abundance, we examined the planktonic growth of bacteria in nitrogen-free minimal medium K. The results showed that neither the individual cultures of *S. stutzeri* A1501 and *B. velezensis* BRI3 nor their interaction system exhibited detectable proliferation within 48 h, which was similar to the nitrogen limited culture conditions (Fig.S1A). Subsequently, we measured the biofilm formation in each culture group at various time points over the 48 h period. Under static conditions, the biofilms of the A1501 culture and the interaction system gradually accumulated and increased in mass with extended incubation time. These results indicated that under nitrogen limited conditions, the presence of BRI3 promotes the transition of A1501 cells from a planktonic state to the biofilm state (Fig. S1B and C).

To gain a deeper understanding of the gene expression levels related to nitrogen fixation and biofilm formation in A1501 during the interaction and to elucidate the potential role of BRI3, we determined the expression levels of the corresponding genes under conditions in which nitrogen-fixing biofilms were formed during the interaction process (Fig. 1C and D). An elevated intracellular concentration of c-di-GMP is critical for bacterial biofilm formation. The *sadC* gene, which encodes the diguanylate cyclase (DGC) that synthesizes the second messenger c-di-GMP, was upregulated >7-fold, representing the most significant increase among all tested genes. Additionally, the nitrogen fixation-related genes of A1501 were also significantly upregulated following the interaction, with the most notable increases observed in *nifD* (encoding the α-subunit of nitrogenase molybdenum-iron protein) and *nifK* (encoding nitrogenase reductase), both of these genes, exhibited more than a 5-fold upregulation. These findings demonstrate that the introduction of BRI3 markedly augments both the nitrogen-fixing capacity and the biofilm-forming ability of A1501, thereby providing a robust foundation for its rhizosphere colonization and subsequent plant growth-promoting functions.

### Surfactin synthesized by *B. velezensis* BRI3 mediated the biofilm formation of *S. stutzeri* A1501

**Fig 2:**
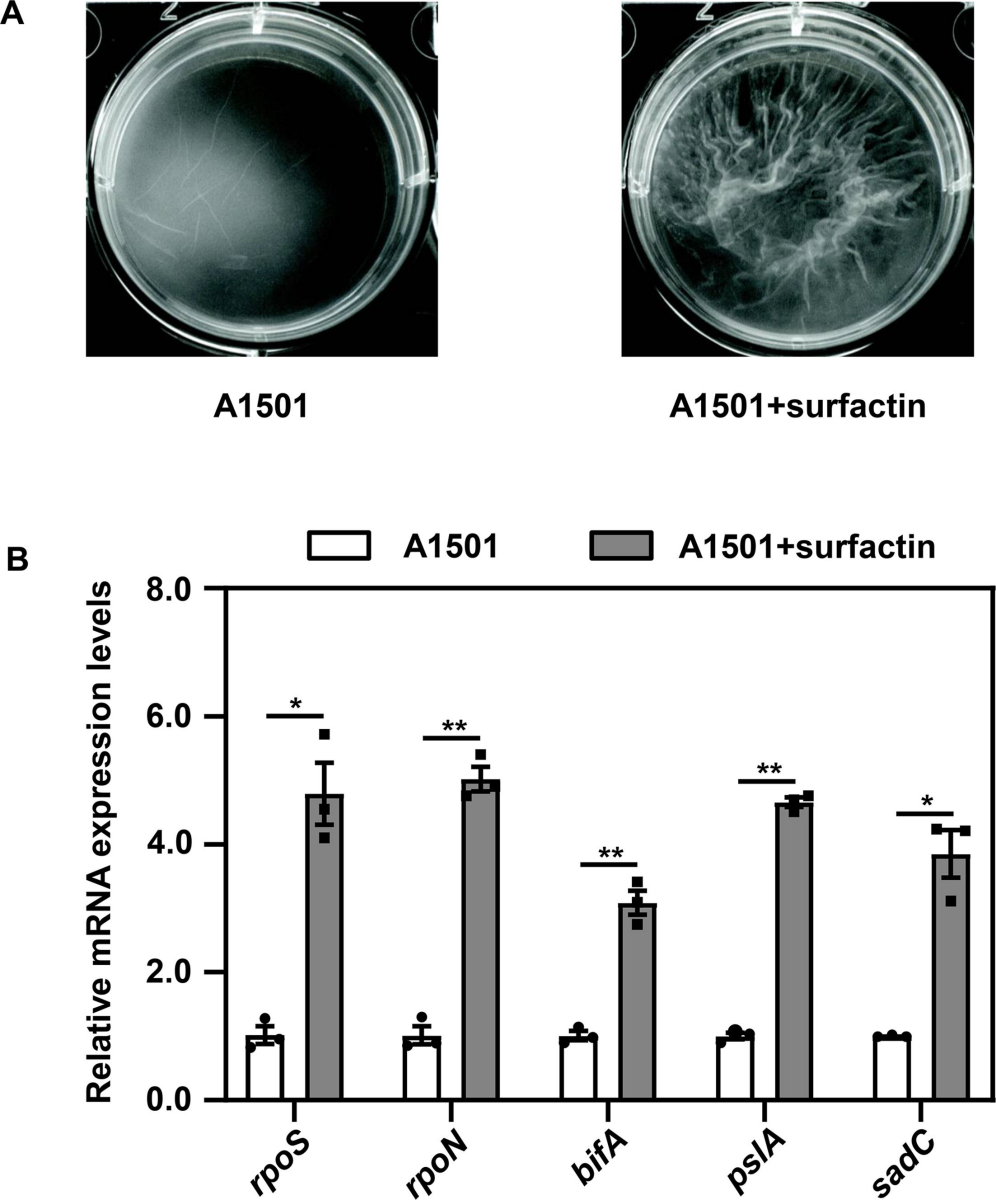
Surfactin enhances the biofilm formation of *S. stutzeri* A1501 by *B. velezensis* BRI3. (**A**) Formation of mature biofilms by *S. stutzeri* A1501 after 48 h of cultivation, either in monoculture or surfactin-supplemented (0.4 mg/mL) culture, as observed in 6-well plates: bacteria were inoculated in minimal medium K, which lacked a nitrogen source and contained carbon substrates at 50 mM. (**B**) Relative mRNA expression levels of genes related to biofilm formation in nitrogen-fixing biofilm cells under monoculture or surfactin-supplemented (0.4 mg/mL) culture conditions. The data are presented as the mean ± s.d. (*n* = 3). The significance test was performed using a t test. * indicates a significant difference (*P* ≤ 0.05); ** indicates a highly significant difference (*P* ≤ 0.01).

Our previous study confirmed through genomic analysis that BRI3 harbors a complete surfactin biosynthetic gene cluster (*srfAA-AD* operon) (34). LC-QTOF MS/MS analysis revealed that the fragment ion peaks of the major bioactive substance in BRI3 culture were completely consistent with those of commercial surfactin standard, confirming the surfactin-producing capability of BRI3 (Fig. S2). To investigate whether surfactin contributes to the enhanced biofilm formation of *S. stutzeri* A1501, we evaluated the effect of exogenous surfactin on biofilm development. We employed nitrogen-free minimal medium K to assess the ability of different systems to form mature biofilms after 48 h of inoculation (Fig. 2). The results showed that under nitrogen-limited conditions, biofilms formed under the condition of A1501 monoculture were relatively sparse. In contrast, the addition of 0.4 mg/mL surfactin led to the formation of significantly more mature and compact biofilms (Fig. 2A). Other concentrations of surfactin also improved the biofilm-forming ability of A1501 to varying degrees (Fig. S3).

To further clarify the regulatory mechanism underlying this promotion effect, we analyzed the relative mRNA expression levels of several key biofilm-related genes in A1501, including *rpoS* (encodes the stress-response sigma factor σ^S^, which regulates bacterial biofilm formation), *rpoN* (encodes the alternative sigma factor σ⁵⁴, which controls bacterial nitrogen assimilation), *bifA* (encodes a c-di-GMP-specific phosphodiesterase), *pslA* (encodes a glycosyltransferase essential for the biosynthesis of Psl exopolysaccharide), and *sadC*, under both monoculture and surfactin-supplemented culture conditions. Compared with the monoculture conditions, surfactin supplementation significantly upregulated the expression of all these biofilm-associated genes. Notably, the expression of the *rpoN* gene was increased the most, with more than 5-fold upregulation (Fig. 2B). Taken together, these results demonstrate that surfactin produced by *B. velezensis* BRI3 promotes biofilm formation in *S. stutzeri* A1501 by upregulating multiple biofilm-related genes.

### Microbial synergy with *B. velezensis* BRI3 promoted the rhizosphere colonization capability of *S. stutzeri* A1501

**Fig 3:**
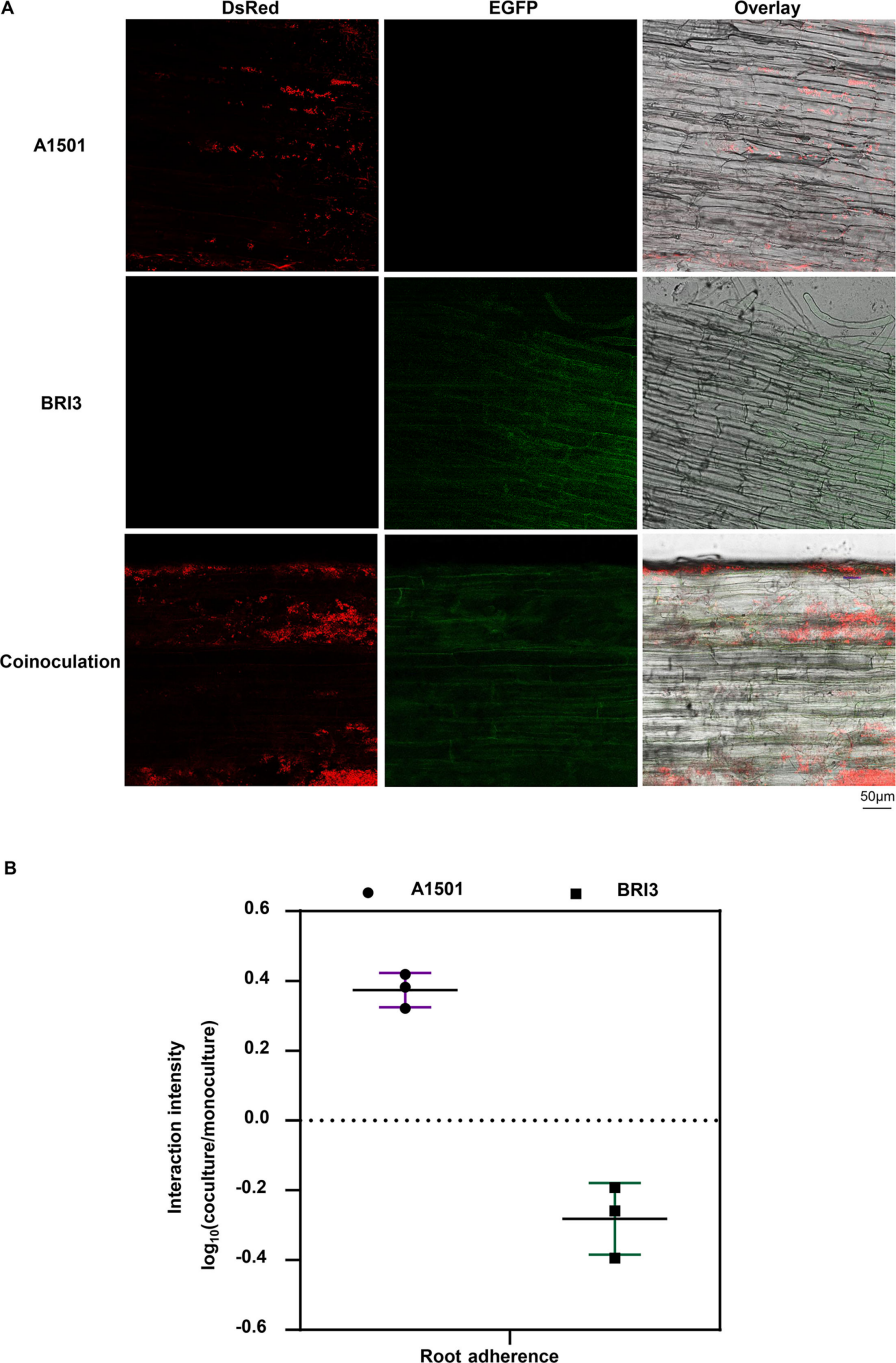
Maize root adherence by *S. stutzeri* A1501 and *B. velezensis* BRI3. (**A**) Maize roots were colonized by *S. stutzeri* A1501 pL*rfp* (DsRed, colored red) and *B. velezensis* BRI3 pL*egfp* (EGFP, colored green), and the mixture was visualized using CLSM. (**B**) The colony-forming ability of the bacteria was measured as CFUs per gram of root (*n* = 3). Interaction intensity is defined as the logarithmic scale of cell numbers during cocolonization relative to the average cell numbers during monocolonization. Interaction intensity >1 indicates facilitation, whereas <1 indicates inhibition. The bars represent the mean ± s.d. values.

Under nutrient-sufficient culture conditions, *B. velezensis* BRI3 promoted the growth of *S. stutzeri* A1501. The synergistic interaction between A1501 and BRI3 under co-culture conditions is shown in Fig. S4. To clarify the relationship between the two strains in the rhizosphere environment, we evaluated the adhesion ability of *S. stutzeri* A1501 and *B. velezensis* BRI3 on maize roots to determine their colonization potential (Fig. 3). To distinguish between the two strains and enable dynamic observation, fluorescently labeled engineered strains were employed. Specifically, *S. stutzeri* A1501 (pL*rfp*) expresses red fluorescent protein (RFP), and *B. velezensis* BRI3 (pL*egfp*) expresses enhanced green fluorescent protein (eGFP). Confocal laser scanning microscopy revealed that these two strains can colonize the maize rhizosphere. Compared with monoinoculation, coinoculation increased the proportion of the maize rhizosphere colonized by A1501 (Fig. 3A). The proportion of the maize rhizosphere colonized by each strain was determined by plate counting. The results indicated that under coinoculation conditions, A1501 emerged as the dominant species in the population colonizing the maize rhizosphere (Fig. S5). The root adherence results, expressed as interaction intensity, indicate that the colonization of A1501 is promoted under coinoculation conditions, whereas that of BRI3 is suppressed (Fig. S5 and Fig. 3B). These results are consistent with the above research findings, both indicating the predominant role of A1501 in shaping cooperation.

### Coinoculation with *S. stutzeri* A1501 and *B. velezensis* BRI3 promoted the growth of maize

First, we assessed the biosynthesis of certain plant growth-regulating hormones. Our experimental results showed that *S. stutzeri* A1501 and *B. velezensis* BRI3 are both capable of producing indole acetic acid (IAA), exopolysaccharide (EPS), and gibberellic acid (GA). When cultured together, the two strains synthesized more IAA and EPS. The increased EPS production may be closely associated with the enhanced biofilm formation, while it is more noteworthy that coculture significantly increased IAA synthesis 47.3% higher than that in the monoculture of A1501. This finding suggests that the combination of these two strains may be more conducive to plant growth. In contrast, there was no significant difference in GA production among all groups. We also evaluated siderophore production to further clarify the interspecific interactions between the two strains. *S. stutzeri* A1501 can produce siderophores, but BRI3 cannot. Furthermore, the coculture system did not significantly promote siderophore production (Table 1).

**TABLE 1:** *In vitro* plant growth-promoting characteristics of *S. stutzeri* A1501 and *B. velezensis* BRI3 in the coculture system^*a*^

Growth-promoting indicators	Treatment		
	A1501	BRI3	A1501 + BRI3
Indole acetic acid (IAA) (μg/mL)	37.63 ± 1.20 b	15.01 ± 0.93 c	55.43 ± 1.37 a
Exopolysaccharide (EPS) (g/L)	0.1778 ± 0.0023 b	0.1780 ± 0.0033 b	0.1879 ± 0.0029 a
Siderophore (degradation zone diameter/cm)	1.78 ± 0.03 a	0.00 ± 0.00 b	1.70 ± 0.10 a
Gibberellic acid (GA) (pmol/L)	127.80 ± 15.12 a	129.24 ± 13.55 a	128.88 ± 11.58 a

^*a*^ Values in the same row followed by different lowercase letters (a, b, c) indicate significant differences (P ≤ 0.05), as determined by one-way analysis of variance (ANOVA); values sharing the same letter are not significantly different. All data are presented as mean ± SD (n = 3).

**Fig 4:**
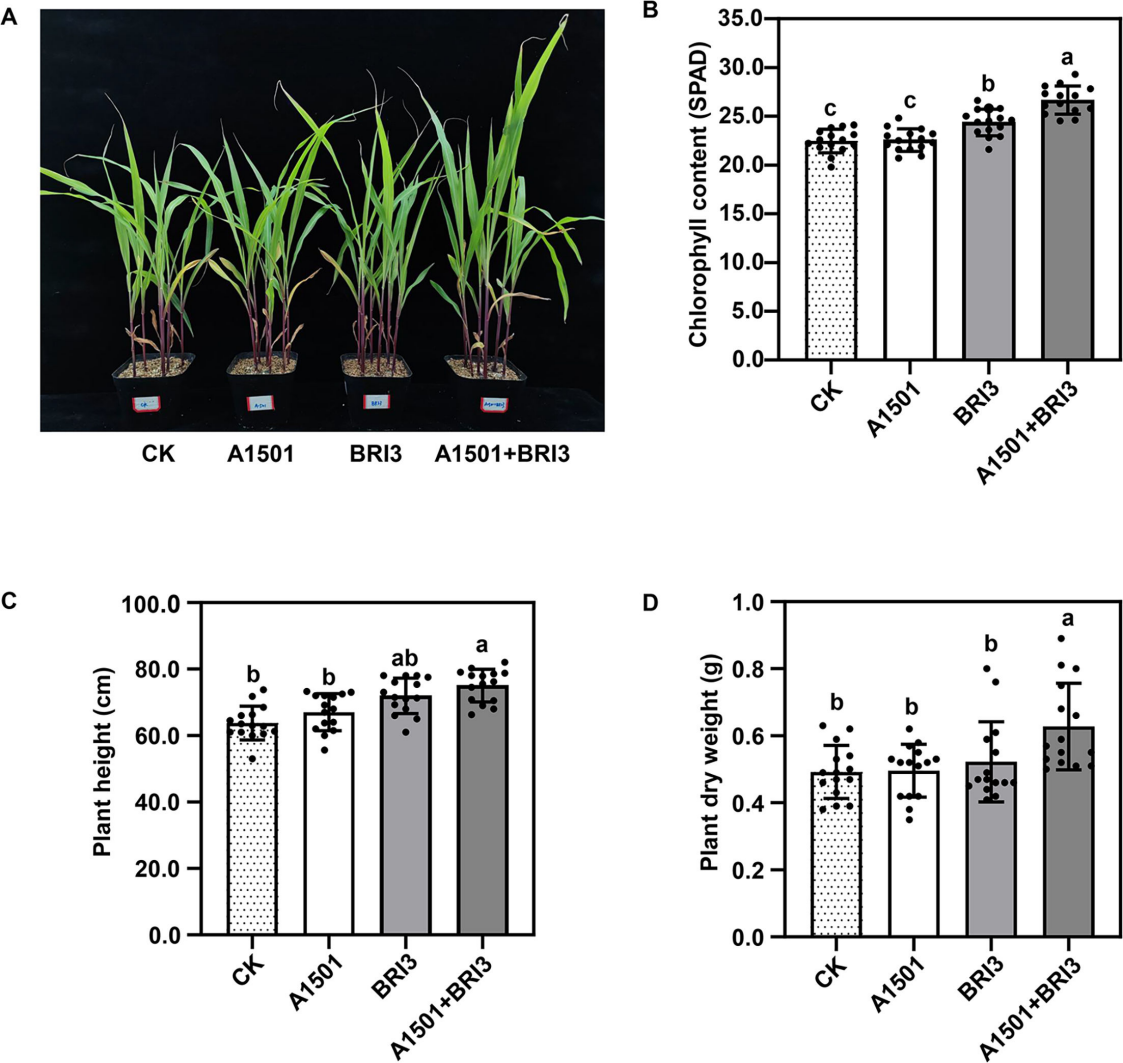
The microbial synergy of *S. stutzeri* A1501 and *B. velezensis* BRI3 promoted plant growth. (**A**) Four-week-old maize plants were grown in sterilized vermiculite inoculated with *S. stutzeri* A1501, *B. velezensis* BRI3 or both *S. stutzeri* A1501 and *B. velezensis* BRI3 (a mixture of two strains). CK represents plants grown in ordinary soil without inoculation. (**B**) Leaf chlorophyll content of the plants. (**C**) Height of the plants. (**D**) Dry weight of the plants. The bars represent the mean ± s.d. (*n* = 15). Significance tests were performed using one-way ANOVA followed by Tukey’s *post hoc* test via Prism 8. Different letters indicate statistically significant (*P* ≤ 0.05) differences.

We further evaluated whether the microbial synergy of the two species outperformed that of the individual strains in a maize pot experiment. Both strains promoted the growth of maize under monoinoculation and coinoculation conditions (Fig. 4). Remarkably, coinoculation of *S. stutzeri* A1501 and *B. velezensis* BRI3 more strongly promoted maize growth in comparison to maize inoculated with only one strain. Coinoculation increased the chlorophyll content by 28.78%, plant height by 11.94%, and plant dry weight by 26.61%. Compared with monoinoculation with BRI3, coinoculation significantly increased the chlorophyll content by 23.41%, plant height by 4.30%, and plant dry weight by 20.15%.

To elucidate the growth-promoting effects of *S. stutzeri* A1501 and *B. velezensis* BRI3 coinoculation on maize, we conducted field experiments in two experimental fields located in Qiqihar, Heilongjiang Province, and Dongying, Shandong Province, China. Two soil nutrient conditions, namely, full nitrogen fertilization (100% nitrogen) and 15% reduced nitrogen fertilization (85% nitrogen), were used to fully elucidate the effects of the coinoculation of the two strains under nitrogen-limited conditions. The growth parameters of the maize are shown in Table 2. Monoinoculation with A1501 did not significantly promote yield in the field in Qiqihar but resulted in a 3.3% increase in yield in Dongying. In contrast, monoinoculation with BRI3 resulted in a more pronounced yield-promoting effect on maize (> 5.6%). Interestingly, in the soil with a 15% nitrogen reduction, the maize yield with coinoculation of the two strains was not only significantly greater than that with monoinoculation (*P* ≤ 0.05) but also exceeded the yield of the full nitrogen nutrition group by more than 11.3%, effectively compensating for the 15% nitrogen deficiency while also promoting increased productivity. In addition, compared with monoinoculation, coinoculation with A1501 and BRI3 promoted the growth of other indices of maize, such as plant height, ear height, and ear weight.

**TABLE 2:** Effects of *S. stutzeri* A1501 and *B. velezensis* BRI3 coinoculation on growth parameters of maize plants^*a*^

Treatment	Qiqihar, Northeast China							Dongying, Yellow River Delta, China						
	Plant height (cm)	Ear height (cm)	Ear dry weight (kg)	Number of kernels per ear	100- kernel dry weight (g)	Grain yield (10^3^ kg/ha)	Increase (%)	Plant height (cm)	Ear height (cm)	Ear dry weight (g)	Number of kernels per ear	100- kernel dry weight (g)	Grain yield (10^3^ kg/ha)	Increase (%)
100% Nitrogen	329 ± 3 bc	120 ± 1 a	0.27 ± 0.01 b	250.50 ± 8.12 b	42.0 ± 0.3 c	7.1 ± 0.1 b	0	229 ± 1 d	103 ± 2 d	95.00 ± 2.83 b	208.50 ± 4.21 b	28.7 ± 0.9 b	3.0 ± 0.10 b	0
85% Nitrogen	324 ± 2 ab	121 ± 2 a	0.24 ± 0.01 a	230.34 ± 9.54 a	40.0 ± 0.4 a	6.2 ± 0.2 a	−12.7	202 ± 2 a	81 ± 2 a	85.70 ± 2.87 a	186.00 ± 3.15 a	26.7 ± 0.8 a	2.5 ± 0.09 a	−17.0
85% Nitrogen + A1501	321 ± 2 a	118 ± 2 a	0.26 ± 0.02 ab	255.72 ± 7.21 b	41.1 ± 0.1 b	7.1 ± 0.2 b	0	206 ± 1 b	84 ± 2 a	94.90 ± 2.88 b	205.50 ± 3.18 b	29.7 ± 0.9 bc	3.1 ± 0.07 b	3.3
85% Nitrogen + BRI3	338 ± 3 d	127 ± 2 b	0.27 ± 0.01 b	268.36 ± 4.79 c	41.7 ± 0.2 c	7.5 ± 0.1 c	5.6	228 ± 2 d	95 ± 2 c	96.10 ± 2.70 b	209.50 ± 6.21 b	30.7 ± 0.8 c	3.3 ± 0.06 c	10.0
85% Nitrogen + A1501+BRI3	334 ± 3 cd	131 ± 3 b	0.28 ± 0.01 b	279.13 ± 6.29 d	42.1 ± 0.4 c	7.9 ± 0.1 d	11.3	224 ± 1 c	87 ± 1 b	97.70 ± 2.06 b	212.00 ± 5.53 b	32.1 ± 0.9 d	3.4 ± 0.09 c	13.3

^*a*^ Values in the same column followed by different lowercase letters (a, b, c, d) indicate significant differences (P ≤ 0.05), as determined by Duncan’s multiple range test; values sharing the same letter are not significantly different. All data are presented as mean ± SD (n = 30).

## DISCUSSION

In this study, we reveal a previously unreported microbial synergy between *S. stutzeri* A1501 and *B. velezensis* BRI3 and provide evidence that this partnership greatly increases the nitrogen-fixing capacity of A1501. This microbial synergy with BRI3 upregulates nitrogen-fixation and biofilm-formation genes in A1501, thereby enhancing nitrogen-fixing biofilm formation and rhizospheric competitiveness. Accordingly, the marked increase in nitrogen fixation and rhizosphere colonization capacity caused by this microbial synergy resulted in a significant increase in maize yield under field conditions. For the first time, we propose that surfactin plays a crucial role in this microbial synergy. The discovery of this microbial synergy will facilitate a deeper understanding of cooperative behaviors among microorganisms in the environment and provide guidance for agricultural development and food production.

Our laboratory has previously demonstrated that root-associated *S. stutzeri* A1501 displays a distinctive hierarchy of carbon-source utilization in the rhizosphere (35). Its RNA chaperone Hfq harbors hundreds of target RNAs that orchestrate nitrogen fixation, carbon catabolism, and biofilm formation, collectively conferring exceptional environmental adaptability to this strain (36). In this study, we demonstrate that the incorporation of *B. velezensis* BRI3 can promote the formation of nitrogen-fixing biofilms and increase the nitrogen fixation capacity of *S. stutzeri* A1501. The formation of biofilms through microbial cooperation is among the principal characteristics of beneficial microbial interactions in the plant rhizosphere, which can protect the host from pathogen infection and promote microbial colonization and plant growth-promoting functions (37, 38). The promotional effect of *Bacillus* spp. on the biofilm formation of *Pseudomonas* spp. has been reported in previous studies. *B. velezensis* SQR9 can recruit *Pseudomonas* spp. to the rhizosphere and promote the formation of biofilms (28). During synergistic biofilm formation, the two strains cooperate through metabolic cross-talk (28), which indicates that cooperative interactions between *Bacillus* spp. and *Pseudomonas* spp. are common and that metabolic cross-talk is the main cause of cooperation. In this study, surfactin, the major metabolite of BRI3, is proposed to be involved in stimulating the biofilm formation of A1501, providing compelling evidence for the metabolic cross-talk between these two types of bacteria, a finding that has not been reported previously. We speculate that surfactin, as a compound with inherent biocontrol functions (39–42), may have established a stress environment for the survival of A1501, thereby stimulating this strain to form biofilms for self-protection. Although corresponding phenotypic evidence is currently available, the underlying molecular mechanism remains unknown. In addition to the cross-talk of metabolites, Díaz proposed that *Azospirillum baldaniorum* Sp245 can use the mixed biofilm scaffold or matrix formed with *Pseudomonas fluorescens* A506 to promote its growth, thereby benefiting from a mixed community (43). Therefore, these findings indicate that in addition to exchanging metabolites such as surfactin to stimulate biofilm formation, *S. stutzeri* A1501 and *B. velezensis* BRI3 may also exert promotional effects through other mechanisms, such as the use of scaffolding structures within the mixed system, thereby manifesting the joint formation of nitrogen-fixing biofilms.

Notably, this study emphasizes the cooperative formation of nitrogen-fixing biofilms by the two strains under nitrogen-limiting conditions, with the nitrogen-fixation capacity of *S. stutzeri* A1501 also being enhanced. There have been extensive reports on the phenomena and mechanisms of enhanced nitrogen fixation through microbial interactions within communities. Yang et al. reported that riboflavin synthesized by helper bacteria in the soil may act as a prebiotic to promote nitrogen fixation by nitrogen-fixing bacteria (44). *Bacillus* spp. can synthesize riboflavin (26, 45), suggesting that in this study, *B. velezensis* BRI3 may also increase the nitrogen fixation ability of *S. stutzeri* A1501 through the synthesis and secretion of riboflavin. Another mechanism that may be involved pertains to the utilization of oxygen. *S. stutzeri* A1501 requires a microoxic environment to perform nitrogen fixation (46). Berne et al. reported that oxygen availability regulates the toxin-antitoxin system ParDE4 in bacterial biofilms. ParDE4 stimulates cell death in low-oxygen regions of the biofilm, leading to extracellular DNA (eDNA) release that promotes the dispersal of newborn cells and their colonization of new, more favorable environments (47). This insight prompted us to consider that BRI3 may facilitate the formation of a denser biofilm environment for A1501 through surfactin, and the resulting oxygen-limited conditions may be conducive to the nitrogen-fixing function of A1501, which could be a key factor in the enhanced nitrogen-fixing capacity.

Rhizosphere colonization by PGPR is an essential prerequisite for their beneficial functions (48). Effective biofilm formation is essential for successful rhizosphere colonization and the manifestation of plant growth-promoting properties (49). Research has shown that a consortium consisting of *Xanthomonas*, *Stenotrophomonas*, and *Microbacterium* spp. exhibits enhanced biofilm formation relative to each species in isolation, thereby increasing their beneficial effects on *Arabidopsis* (37). Another report indicated that coinoculation with a rhizobacterial community and an ectomycorrhizal fungus significantly increased the colonization rate of ectomycorrhizal fungi in the plant rhizosphere. Consequently, significant plant growth-promoting effects were observed (50). The results of the present study revealed that *B. velezensis* BRI3 can promote the formation of nitrogen-fixing biofilms of *S. stutzeri* A1501, and the colonization patterns of the two strains in the rhizosphere of maize were further investigated. In the context of coinoculation, A1501 exhibited enhanced colonization efficiency in the maize rhizosphere, whereas BRI3 demonstrated the opposite outcome. These findings are consistent with the results concerning the formation of nitrogen-fixing biofilms. This phenomenon has not been previously reported in the literature on the interactions between *Pseudomonas* spp. and *Bacillus* spp.

Finally, the coinoculation of *S. stutzeri* A1501 and *B. velezensis* BRI3 promoted plant growth, as observed in both our pot experiments and field trials. This growth-promoting effect is also reflected in the expression of growth-promoting indicators such as IAA and EPS. Similar studies have demonstrated that inoculation with *B. megaterium* CNPMS B119 and *B. subtilis* CNPMS B2084 significantly increases phosphorus solubilization in the soil, thereby increasing phosphorus uptake by maize (51). The increased phosphorus acquisition directly promotes plant growth and results in higher maize yields. Another study reported that coinoculation with *Pseudomonas* spp. and *Bradyrhizobium ottawaense* effectively enhanced nodule formation and nitrogen fixation, thereby significantly improving the growth and yield of Japanese soybean cultivars (52). These results collectively demonstrate the significant application potential of the coinoculation of PGPR in promoting crop growth. Notably, the growth-promoting capacity demonstrated in this study is predicated on nitrogen-deficient conditions. The nitrogen fixation capacity of A1501 has been established (46), and its plant growth-promoting ability in maize has also been evaluated (23). Inspired by the roles of *S. stutzeri* A1501 and *B. velezensis* BRI3 in rhizosphere colonization and nitrogen-fixing biofilm formation, we determined that *B. velezensis* BRI3 can promote plant growth by enhancing the nitrogen fixation ability of *S. stutzeri* A1501. This discovery underscores the substantial potential of these two strains for biofertilizer formulation.

Our study yielded several notable experimental findings and highlighted some technical considerations, which merit in-depth discussion to further elucidate the underlying regulatory mechanisms. In the previous reports of c-di-GMP signaling regulation, a concurrent upregulation of the *sadC* and *bifA* genes was observed in the interaction system. Accumulating evidence has demonstrated that intracellular c-di-GMP homeostasis in bacteria is synergistically maintained by diguanylate cyclases (DGCs) and c-di-GMP-specific phosphodiesterases (PDEs), which collectively govern the transition between planktonic and biofilm phenotypes—a conserved regulatory strategy prevalent across diverse bacterial species (53). In this study, the simultaneous upregulation of *sadC* and *bifA*, which encode functionally antagonistic enzymes, is not a paradoxical observation but rather a manifestation of the sophisticated spatiotemporal regulation of intracellular signaling networks in response to interspecific interactions. Specifically, the substantial (>7-fold) upregulation of *sadC* facilitates a rapid elevation of intracellular c-di-GMP levels, which serves as a pivotal trigger for biofilm initiation and maturation. In contrast, the moderate (~2-fold) upregulation of *bifA* functions as a negative feedback buffer, preventing the irreversible bacterial sessility that would result from excessive intracellular c-di-GMP accumulation. Furthermore, as previously documented (54, 55), metabolic energy cost represents a critical factor modulating this regulatory process: DGC-mediated c-di-GMP synthesis is energetically costly, as it consumes high-energy GTP equivalents, thereby necessitating high-level transcriptional upregulation to drive phenotypic switching. In contrast, PDE-mediated c-di-GMP hydrolysis is energetically efficient, involving only phosphodiester bond cleavage, such that moderate upregulation is sufficient to achieve effective signal attenuation without squandering cellular resources. This finding extends beyond our initial hypothesis of *sadC*-only upregulation, revealing that interspecific crosstalk can globally reprogram the behavioral regulatory circuitry of diazotrophic bacteria.

In the coculture interaction assay, we observed that the growth of strain BRI3 was inhibited to a certain extent under laboratory conditions. This phenomenon mainly resulted from nutrient competition and metabolite accumulation in the closed culture system, representing a specific phenotype under the given experimental conditions. As reported by Hibbing et al. (56), the strong competitive inhibition observed with laboratory pure culture often fails to recapitulate the balanced microbial interactions that occur in complex natural environments such as agricultural soils and the rhizosphere. The rhizosphere provides abundant resources, open ecological niches, and a complex microbial community, which collectively mitigate pairwise competition and promote more balanced interspecies relationships (57). Therefore, the inhibitory phenotype observed under laboratory conditions does not fully represent the actual interactions between strains in natural environments, and the interpretation of the results should take into account the impact of environmental differences. Notably, our study also revealed that BRI3 still exerted a positive growth-promoting effect on A1501 even in the laboratory culture system.

Another noteworthy experimental observation was that when measuring the growth curves of monocultures vs interaction systems, the OD_600_ exhibited a nonlinear relationship with the actual bacterial concentration. Specifically, when two bacterial strains, each adjusted to an initial OD_600_ of 0.1, were mixed, the OD_600_ value of the resulting co-culture was not twice that of the individual pure cultures. The mechanism underlying the nonlinear relationship between OD_600_ absorbance and bacterial concentration has been documented (58, 59). The primary underlying reason is the variation in cell diameter among different strains (60); furthermore, even within the same bacterial species, cell dimensions can shift under different physiological states (61). Since cell diameter substantially amplifies light scattering effects, such variations introduce systematic deviations in OD_600_ measurements, ultimately disrupting the ideal linear relationship between absorbance and bacterial concentration.

This study demonstrates that surfactin can directly stimulate biofilm formation in A1501, yet this conclusion remains to be validated at the genetic level. To address this limitation, we attempted to construct a surfactin synthesis gene mutant of BRI3 via homologous recombination; however, multiple attempts failed to establish a stable genetic manipulation system for this strain. The unsuccessful construction of the BRI3 mutant is presumably attributable to a highly active restriction-modification system, low cell membrane permeability, and the lack of strain-specific optimization of transformation parameters—all of which represent common technical bottlenecks in environmental microbiology research (62–65). To address this challenge, genome editing technologies such as CRISPR-Cas9 may be employed in future studies as alternatives to traditional methods. In addition, the technical barriers in establishing the genetic manipulation system of BRI3 may be circumvented via the optimization of cell membrane permeability and transformation system parameters (66, 67). Future successful establishment of such a genetic manipulation system would enable the construction of BRI3 mutants deficient in surfactin synthesis and biofilm regulation-related genes, thereby elucidating the underlying regulatory mechanisms. This would also provide crucial technical support for the genetic improvement of the BRI3 strain and its application in agriculture.

In summary, our study demonstrated that *B. velezensis* BRI3 increases the nitrogen fixation and rhizosphere colonization capabilities of *S. stutzeri* A1501, thereby increasing nitrogen availability and significantly improving the growth and yield of maize. *S. stutzeri* A1501 and *B. velezensis* can be applied as coinoculants in agricultural production. Our study provides an ecological approach to increase the yield of maize under nitrogen-limited conditions through bacterial interactions, which also offers new insights into the modes of plant rhizosphere microbial interactions.

## MATERIALS AND METHODS

### Bacterial strains and growth conditions

The bacterial strains used in this study are meticulously listed (Table S1). All the strains involved were grown in lysogenic broth (LB) or minimal medium K (containing 0.4 g L^−1^ KH_2_PO_4_, 0.1 g L^−1^ K_2_HPO_4_, 0.1 g L^−1^ NaCl, 0.2 g L^−1^ MgSO_4_·7H_2_O, 0.01 g L^−1^ MnSO_4_·H_2_O, 0.01 g L^−1^ Fe_2_(SO_4_)_3_·H_2_O, and 0.01 g L^−1^ Na_2_MoO_4_·H_2_O, pH 6.8) supplemented with sodium lactate (50 mM) as the sole carbon source and no nitrogen source (22). On the basis of different experimental objectives, the bacterial strains were incubated at a constant temperature of 30°C under either shaking conditions at 220 rpm or static conditions.

### Biofilm formation assays

The formation of surface-adhered biofilms was assessed through the crystal violet method in 96-well PVC plate (Corning). *S. stutzeri* A1501 and *B. velezensis* BRI3 were grown overnight in LB at 30°C. The cultured bacteria were centrifuged, and the supernatant was removed. To assess the ability of bacteria to form nitrogen-fixing biofilms, biofilm formation assays were conducted using minimal medium K. The collected cells were resuspended in fresh minimal medium K containing sodium lactate (50 mM) without nitrogen sources, adjusting the final OD₆₀₀ to 0.1. The adjusted culture was dispensed into separate wells of a 96-well PVC plate, ensuring a final volume of 200 μL per well. The 96-well PVC plate was sealed with breathable sealing membrane (Sigma-Aldrich) and incubated statically at 30°C for 48 h. During incubation, samples were taken every 12 h, and the liquid medium as well as non-adherent planktonic cells in the wells were carefully aspirated from the PVC wells using a micropipette. Each well was subsequently washed twice with 150 μL of sterile double-distilled water. Each well was treated with 150 μL of 0.1% crystal violet solution in ethanol, allowed to stand for 10 min and then washed twice with 200 μL of sterile double-distilled water. Following imaging, the biofilm-associated crystal violet was solubilized with 30% acetic acid, and the resulting solution was quantified by measuring the OD_560_ using the spectrophotometer.

The experiments were divided into three groups: A1501 monoculture, BRI3 monoculture, and interaction system. All groups were cultured under the same conditions, with the only difference being that the monoculture groups were inoculated with a single bacterial strain, while the interaction system was inoculated with both strains at a volume ratio of 1:1. Unless otherwise specified, all subsequent experiments adhered to this grouping protocol.

The assessment of the stimulatory effect of surfactin on biofilm formation was conducted using a 6-well microtiter plate. The bacterial culture and final inoculation concentrations were the same as those described above. A gradient of surfactin concentrations was added to the bacterial cultures, with final concentrations set to 0, 0.1, 0.2, 0.4, 1.6, and 3.2 mg/mL.

### Nitrogenase activity assays

Nitrogenase activity was measured by following a previously described acetylene reduction assay (ARA) protocol, with minor modifications (22). The strains were cultured overnight in LB medium and then centrifuged and resuspended in nitrogen-free minimal medium K. The bacterial suspension and nitrogen-free minimal medium K were transferred into flasks (120 mL) to achieve a final volume of 10 mL and a final bacterial concentration of OD_600_ = 0.1. Following 48 h of static incubation at 30°C, the flasks containing the samples were purged with argon gas to remove the air inside. Subsequently, 0.5% oxygen and 10% acetylene were introduced into the culture system, and gas samples (0.25 mL) were collected every 2 h to measure ethylene production. The collected gas samples were analyzed using a gas chromatograph fitted with a flame ionization detector. The ethylene content in the gas samples was determined by reference to an ethylene standard.

### RNA isolation and qRT-PCR assays

The total RNA used for qPCR analysis was extracted from all bacterial cells in the biofilm culture system after 48 h of static incubation at 30°C. Three independent biological replicates were performed for each sample, and three technical replicates were set for each biological replicate in the qPCR assay. Total RNA extraction was performed with an innuPREP RNA Mini Kit according to the manufacturer’s protocol. The extracted total RNA was subjected to reverse transcription into cDNA using random primers in conjunction with a High-Capacity cDNA Reverse Transcription Kit. Polymerase chain reaction (PCR) amplification was subsequently performed utilizing the Power SYBR Green PCR Master Mix on the ABI Prism 7500 Sequence Detection System. In this experimental setup, the 16S rRNA gene served as the internal reference control, and the relative quantification of gene expression was achieved through the application of the comparative threshold cycle (2^−ΔΔCT^) method. The resulting data were carefully analyzed with the ABI PRISM 7500 Sequence Detection System Software. The primers employed were meticulously designed on the basis of the complete genome sequence of *S. stutzeri* A1501 (Table S2).

### LC-QTOF MS/MS assays

The liquid fermentation metabolites of *B. velezensis* BRI3 were extracted and concentrated using rotary evaporation with methanol. The obtained extract was analyzed by liquid chromatography coupled with quadrupole time-of-flight tandem mass spectrometry (LC-QTOF MS/MS). Chromatographic separation was performed on a Poroshell 120 SB-C18 analytical column (2.1 mm × 100 mm, 2.7 µm). Mobile phase A was water containing 0.1% (vol/vol) formic acid, and mobile phase B was chromatographic grade acetonitrile containing 0.1% (vol/vol) formic acid. The flow rate was 0.35 mL/min. The gradient elution program was set as follows: 10 min, 5% A and 95% B; 13 min, 5% A and 95% B; 13.01 min, 90% A and 10% B; 15 min, 5% A and 95% B. Mass spectrometry was operated using a Dual AJS ESI ion source. The full-scan *m*/*z* range was 100–2,000, and the MS/MS *m*/*z* range was 50–2,000. The gas temperature was 300℃, drying gas flow was 8 L/min, sheath gas temperature was 350 ℃, sheath gas flow was 11 L/min, nozzle voltage was 1 kV, and collision energy was 15–50 eV. LC-QTOF MS/MS assays were performed using commercial surfactin standard (Solarbio, purity ≥ 98%) as the reference control.

### Growth curve assays

*S. stutzeri* A1501 and *B. velezensis* BRI3 were cocultured in nitrogen-free minimal medium K. The two strains were separately grown overnight in LB medium and then centrifuged. After discarding the supernatant, the cell pellets were resuspended in nitrogen-free minimal medium K and adjusted to an OD_600_ of 1.0. The suspensions inoculated into 50-mL Erlenmeyer flasks with a working volume of 20 mL, and the initial OD_600_ was adjusted to 0.1. The cultures were incubated at 30°C with shaking at 220 rpm. The OD_600_ value was measured every 6 h using a spectrophotometer. Each treatment was replicated three times.

### Swarm assay

To investigate the discrimination behavior exhibited during the encounter of swarming colonies between A1501 and BRI3, swarm assays were conducted using 9 cm plates containing freshly prepared B medium (containing 2 g L^−1^ (NH_4_)_2_SO_4_, 2 g L^−1^ MgSO_4_ × 7H_2_O, 2 g L^−1^ KCl, 2 g L^−1^ sodium citrate × 2H_2_O, 7.8 g L^−1^ Tris∙HCl, pH 7.5, 0.3 g L^−1^ CaCl_2_ × 2H_2_O, 0.0003 g L^−1^ FeSO_4_ × 7H_2_O, 0.0022 g L^−1^MnSO_4_ × 4H_2_O, 0.08 g L^−1^ KH_2_PO_4_, 0.7 g L^−1^ sodium glutamate, 0.12 g L^−1^ lysine, 0.15 g L^−1^ tryptophan, and 2 g L^−1^ glucose) solidified with 0.7% agar. Strains were inoculated from fresh LB plates into 5 mL liquid B medium and incubated overnight at 37 °C with constant shaking. Overnight cultures were diluted to an optical density of 0.5 at 600 nm (OD_600_), and 2 μL aliquots were spotted onto opposite sides of the agar plates. After drying in a laminar flow hood, the plates were incubated at 37 °C for 48 h and photographed. The phenotypic characteristics of the interacting swarms were assessed based on the captured images (68, 69).

### Quantification of cell numbers by digital PCR

*S. stutzeri* A1501 and *B. velezensis* BRI3 were coinoculated in LB medium at an initial OD_600_ of 0.1 and a volume ratio of 1:1 (to assess bacterial interactions in growing systems, LB medium was employed for interaction-related experiments to ensure normal bacterial growth). After incubation at 30°C for 48 h, the bacterial cells in 1 mL of the medium were harvested. Single-copy genes were selected from the genomes of *S. stutzeri* A1501 and *B. velezensis* BRI3, respectively, and strain-specific primer pairs for digital PCR were designed on the basis of the genomes of A1501 and BRI3 (Table S1). The genomic DNA of A1501 and BRI3 was extracted using an Omega E.Z.N.A. Bacterial DNA Kit and the absolute copy numbers of the target genes were subsequently quantified by the Naica Crystal digital PCR system, which was used to characterize the bacterial cell numbers. Single-copy genes were selected from the genomes of A1501 and BRI3 and were used as target genes to quantify the bacterial cell numbers on the basis of their copy numbers. The absolute copy number of the gene = (copy number of the target gene − copy number of ntc) × 25 µL × dilution factor. The copy number for the ntc was determined by performing PCR amplification in the digital PCR system with ddH_2_O instead of genomic template, which was intended to eliminate the effects of primer dimers and systematic errors, and the reaction system volume is 25 μL.

### Plate counting assays

Bacterial culture was performed according to the method used in the digital PCR assay. A 1 mL aliquot of the bacterial culture was harvested by centrifugation, and the pellet was serially diluted with physiological saline. A 100 μL volume of each dilution was evenly spread onto LB solid medium, with three replicates per dilution. The inoculated plates were incubated at 30℃ for 24 h, followed by colony counting.

### Determination of plant growth-promoting characteristics

The IAA biosynthesis capability of the bacterial strains was quantitatively assessed through the Salkowski colorimetric assay, with spectrophotometric detection at 530 nm (70). EPS production by the bacterial strains was quantified according to the methods of Titus and Ohno with minor modifications (71, 72). Siderophore production was determined using the chrome azurol S assay according to the manufacturer’s protocol. The detection of gibberellins relies on the enzyme-linked immunosorbent assay (ELISA) method provided in the gibberellin detection kit.

### Root colonization assays

The maize rhizosphere colonization experiment was conducted with minor modifications to the method described in previous reports (73). To assess the ability of the strains to colonize the maize rhizosphere, the colonized maize roots were transferred to 50 mL centrifuge tubes and subjected to ultrasonication. The resulting cell suspensions were subjected to viable plate counting via the spread plate method. To calculate the CFU/g of the root, the obtained colony-forming units were divided by the weight of the corresponding root.

### Confocal laser scanning microscopy

The engineered strains and plasmids used in this study were constructed using conventional techniques. To generate an amplicon of the *rfp* gene, appropriate oligonucleotide primers were designed. The amplified *rfp* gene was subsequently cloned and inserted into pBBR1MCS-2 as the vector (74), designated pBBR1MCS-2-RFP. The resulting plasmid was subsequently introduced into A1501 by triparental mating using pRK2013 (75). The plasmid pHY300PLK-EGFP (Miaoling Biological Co., Ltd.), carrying a green fluorescent tag, was introduced into BRI3 cells by electroporation. The strains carrying different fluorescent tags were named A1501 (pL*rfp*) and BRI3 (pL*egfp*). Root colonization at 24 h was visualized with confocal laser scanning microscopy (CLSM). Fluorescent reporter excitation was performed with an argon laser at 490 and 556 nm, and the emitted fluorescence was recorded at 494–550 nm and 560–630 nm for EGFP and DsRed, respectively.

### Greenhouse experimental design

A pot experiment was conducted in the greenhouse of the Biotechnology Research Institute, Chinese Academy of Agricultural Sciences, to evaluate the effects of the strains on maize growth and development. The maize seeds were germinated on agar plates for 5 days and then transferred to vermiculite for a 2-day acclimation period. Different bacterial suspensions were subsequently applied to 1-week-old maize seedlings. The experimental groups included four treatments: CK, maize treated with 10 mL of sterile water (noninoculated control); A1501, maize inoculated with 10 mL of *S. stutzeri* A1501 bacterial suspension (OD_600_ = 1.0); BRI3, maize inoculated with 10 mL of *B. velezensis* BRI3 bacterial suspension (OD_600_ = 1.0); and A1501 + BRI3, maize inoculated with 5 mL of *S. stutzeri* A1501 bacterial suspension and 5 mL of *B. velezensis* BRI3 bacterial suspension (OD_600_ = 1.0). All treated maize plants were maintained at 30°C under a 16 h light/8 h dark photoperiod until they reached 4 weeks of age. The plant height, dry weight, and chlorophyll content were subsequently measured in all treatment groups, with 14–15 replicates per treatment.

### Determination of maize growth parameters and seed yield

Field experiments with maize were conducted at the agricultural experimental station located in Qiqihar, Heilongjiang Province, and in Dongying, Shandong Province. The soil type in Qiqihar is fertile soil, the properties of soil were described as follows: pH, 7.3; NH_4_^+^-N: 1.74 mg/kg; NO_3_^−^-N, 2.02 mg/kg; total N: 1.56 g/kg, organic C: 75.63 mg/kg; and organic matter: 130.39 mg/kg. The soil type in Dongying is saline-alkali soil, the properties of soil were described as follows: pH, 8.48; NH_4_^+^-N: 0.37 mg/kg; NO_3_^−^-N, 5.20 mg/kg; total N: 0.68 g/kg; and organic matter: 8.68 mg/kg. Two nutritional conditions were established for these experiments, including full nitrogen (100% nitrogen, 180 kg ha^-1^) and 15% reduced nitrogen fertilization (85% nitrogen, 153 ha^-1^). Prior to sowing, the seeds of Zhengdan 958 were soaked in suspensions of *S. stutzeri* A1501, *B. velezensis* BRI3, and a mixed bacterial solution, respectively. The planting density in these two fields were about 30,000 plants ha^-1^, with a row-to-row spacing of 70 cm. Crop management was the same as the local maize field. The yield was calculated based on the number of kernels per ear.

### Statistical analysis

Data analysis and figure production were conducted using GraphPad Prism 8. The detailed statistical analysis is described in the figure legends.

## Data Availability

Data will be made available on request.

## SUPPLEMENTAL MATERIAL

Supplemental material aem.00498-26-s0001.pdf Tables S1 and S2; Fig. S1 to S5.

ASM does not own the copyrights to Supplemental Material that may be linked to, or accessed through, an article. The authors have granted ASM a non-exclusive, world-wide license to publish the Supplemental Material files. Please contact the corresponding author directly for reuse.
