# Supplementary material for: A bacterial ally for nitrogen-fixing biofilm: enhancing the rhizosphere colonization of Stutzerimonas stutzeri A1501 with surfactin-producing Bacillus velezensis BRI3
Source: Appl Environ Microbiol. 2026 May 29;92(6):e00498-26. doi: 10.1128/aem.00498-26 (PMC13274376; doi:10.1128/aem.00498-26)
Supplement: Supplemental material — Tables S1 and S2; Fig. S1 to S5. [file aem.00498-26-s0001.pdf]

A bacterial ally for nitrogen-fixing biofilm: Enhancing the rhizosphere colonization of *Stutzerimonas stutzeri* A1501 with surfactin-producing *Bacillus velezensis* BRI3

Supplementary Materials and Additional Experimental Data

Table S1 Strains and plasmids used in this study

Strain/plasmid	Relevant characteristics	Source/reference
**Strain**		
A1501	WT, Chinese culture collection: CGMCC 0351	
A1502, Δ*nifH*	Δ*nifH* deletion mutant	Desnoues et al. (2003)
A1501 (pL*rfp*)	A1501 containing pL*rfp*, Kan^R	This study
BRI3	WT, Guangdong microbial culture collection center: GDMCC 63555	
BRI3 (pL*egfp*)	BRI3 containing pL*egfp*, Tc^R	This study
**Plasmid**		
pHY300PLK-EGFP	An *E. coli* - *Bacillus* spp. shuttle plasmid, Amp^R and Tc^R	Miaoling Biological Co., Ltd.
pRK2013	Helper plasmid for conjugation, Km^R	Lab collection
pBBR1MCS-2	Plasmid for expression RFP fluorescent protein	Lab collection
pBBR1MCS-2-RFP	Plasmid with an RFP fluorescent protein	This study

Table S2 Primers used in this study

Primer*	Sequence (5'-3')	Purpose
rpoN-F	CCAGTAAACCAGCGATCTTGC	qRT-PCR
rpoN-R	CTTCTTCTCCAGCCACGTCAG	qRT-PCR
sadC-F	TGGCGAGTGGTTTCTACGAG	qRT-PCR
sadC-R	ATAGAGCACGAGCAGTGAGC	qRT-PCR
gacA-F	GAAGAGATGGTGCAGGCCA	qRT-PCR
gacA-R	TTTCCCGTTCGGAAAGCAGA	qRT-PCR
bifA-F	GAAGGCGTCGAAACTCCTGA	qRT-PCR
bifA-R	GGCAGGGGCTTGCTGTAATA	qRT-PCR
pslA-F	CTCACCCGAGTTTCGTCGAT	qRT-PCR
pslA-R	ATGATGACGCTGAGTACCGC	qRT-PCR
nifA-F	CGCGAAGACCTCTACTACCG	qRT-PCR, dPCR
nifA-R	CAGCTTGAGTTTGCGACCCT	qRT-PCR, dPCR
nifD-F	ACGACTACGACCGCACCCTC	qRT-PCR
nifD-R	TGGCGAAGCCGTCAAAGC	qRT-PCR

nifH-F	GAGATGATGGCGATGTATGC	qRT-PCR
nifH-R	GGTCGGTGTTGCGGCTGTTG	qRT-PCR
nifK-F	GACAAGGTGGTCGGTAGCA	qRT-PCR
nifK-R	AGCAGCAGGGTGTTCAGG	qRT-PCR
16s rRNA-F	CCTACGGGAGGCAGCAG	qRT-PCR
16s rRNA-R	ATTACCGCGGCTGCTGG	qRT-PCR
Spo0A-F	GATCAGTACAGCGCCCTTAA	dPCR
Spo0A-R	AAGCGTTTCAAACTTCTCG	dPCR
rfp-F	GTCGACGGTATCGATAAGCTT ATGGTGAGCAAGGGCGAGG	Construction of the pBBR1MCS-2-RFP plasmid
rfp-R	CGCTCTAGAACTAGTGGATCC CTACTTGTACAGCTCGTCCATG CC	Construction of the pBBR1MCS-2-RFP plasmid

* F, Forward; R, Reverse.

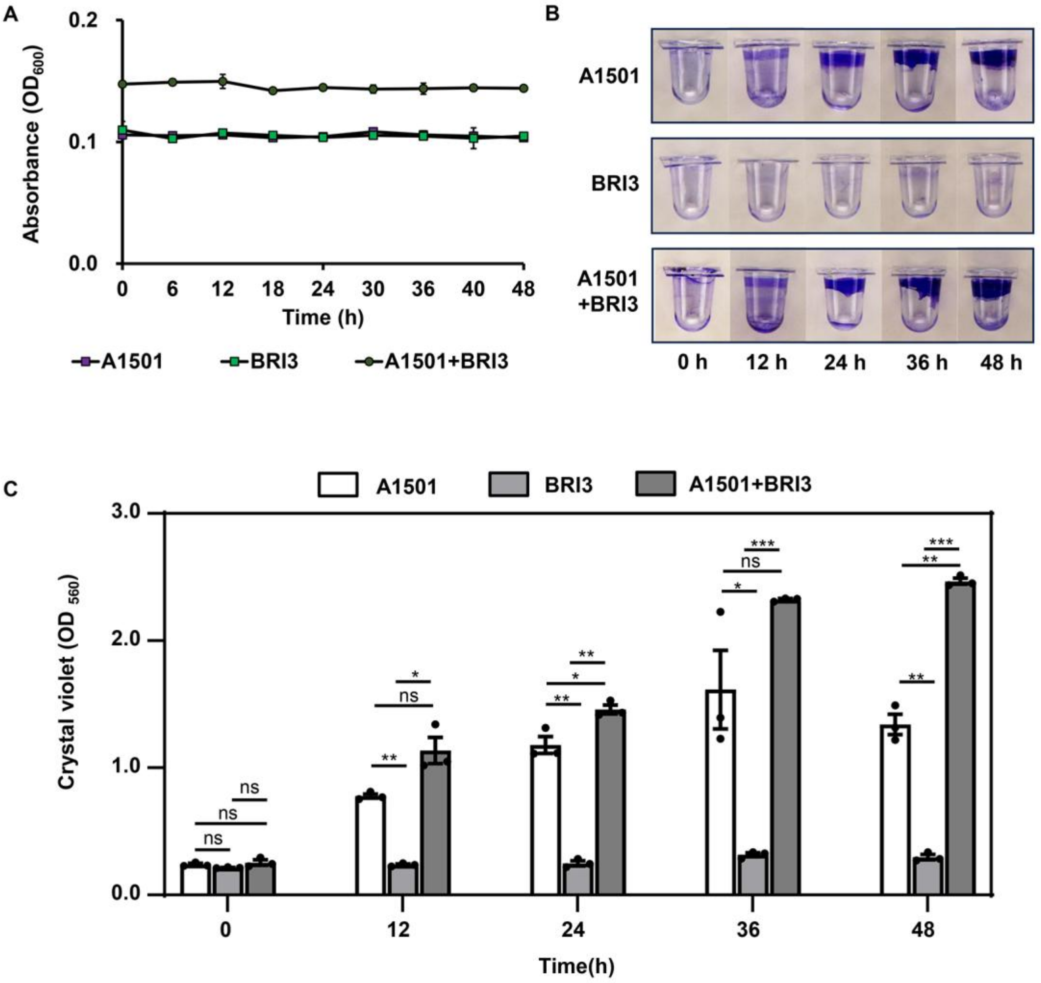

Figure S1 The growth and biofilm formation of *S. stutzeri* A1501 and *B. velezensis* BRI3 Growth curves of *S. stutzeri* A1501 monocultures cultivated in minimal medium K without nitrogen sources (square in purple), *B. velezensis* BRI3 monocultures grown in minimal medium K without nitrogen sources (square in green) and interaction system in the same condition (circle) (panel A). Within the 0 - 48 h incubation period, the biofilm-forming ability of both *S. stutzeri* A1501 monocultures and the corresponding interaction system was found to gradually increase under the culture condition of nitrogen-free minimal medium K. *B. velezensis* BRI3, under the same cultural conditions, did not exhibit any biofilm-forming ability at any tested time point (panel B). The data are presented as the mean ± s.d. (n = 3). The significance test was performed using a t test. * indicates a significant difference ($p \leqslant 0.05$); ** indicates a highly significant difference ($p \leqslant 0.01$); *** indicates a very highly significant difference ($p \leqslant 0.001$); ns indicates no significant difference ($p > 0.05$).

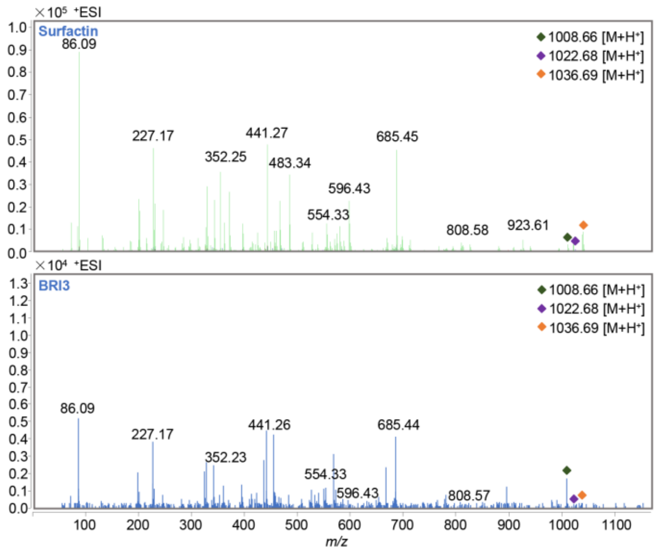

Figure S2 LC-QTOF MS/MS analysis of the bioactive compounds produced by *B. velezensis* BRI3 The LC Q-TOF MS/MS analysis of the surfactin standard and extracts from monoculture of BRI3.

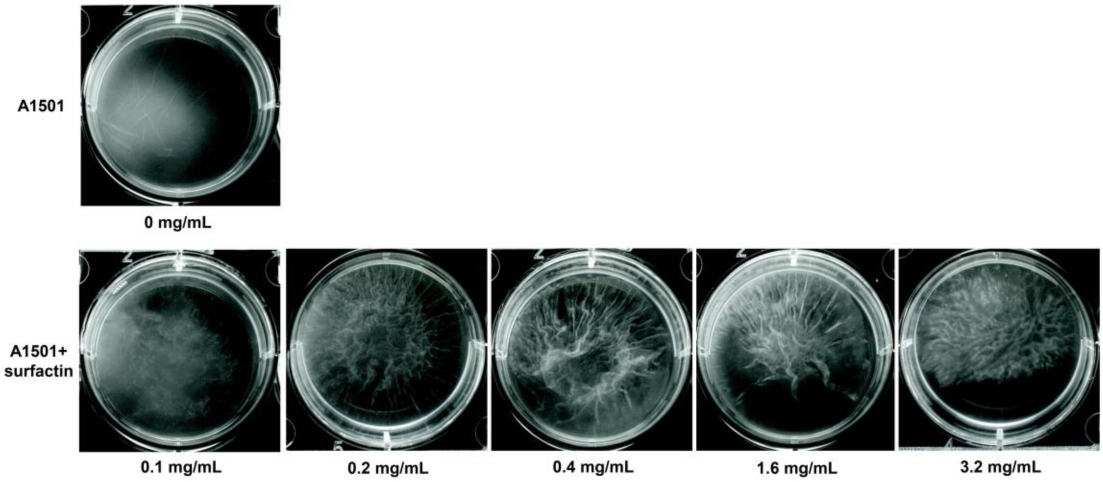

Figure S3 The enhancement of biofilm formation of *S. stutzeri* A1501 by various concentrations of surfactin

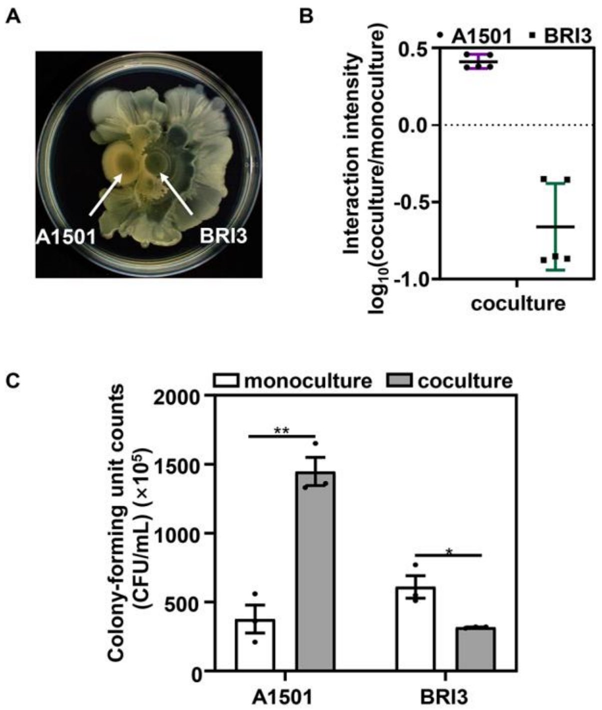

Figure S4 Synergistic interaction between *B. velezensis* BRI3 and *S. stutzeri* A1501 promotes the growth of A1501. The interaction between BRI3 and A1501 under coculture conditions was investigated. Swarming and confrontation assays showed that the two strains approached each other, indicating no antagonism (panel A). The cell numbers of both strains in monoculture and coculture were absolutely quantified to evaluate their growth dynamics. Interaction intensity was calculated as the $\log_{10}$ value of the cell count ratio of coculture relative to monoculture; values > 0 indicate promotion, and values < 0 indicate inhibition. After coculture, the cell count of A1501 was 2.57-fold higher than under monoculture conditions, indicating significant growth promotion, whereas the cell count for BRI3 was reduced to 0.219-fold, indicating growth inhibition (panel B). Plate counting results further confirmed a similar trend: at 48 h, the cell count of A1501 in coculture was 3.84-fold that in monoculture, while that of BRI3 was 0.519-fold. These results suggest that BRI3 contributes to the growth of A1501 in the coculture system. Bars represent the mean ± s.d. (n = 3–5). Statistical significance was assessed via t-test. * indicates significant difference ($p \leq 0.05$); ** indicates highly significant difference ($p \leq 0.01$).

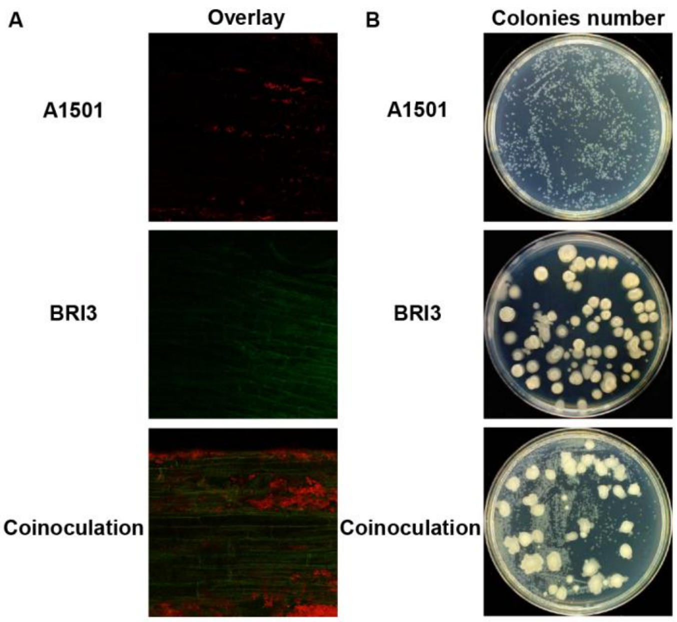

Figure S5 Maize root adherence by *S. stutzeri* A1501 and *B. velezensis* BRI3 The overlay fluorescence images without the bright field were showed in panel A. The colonies of rhizosphere-colonized bacteria on plates were observed, and the two strains exhibited clearly distinguishable morphological characteristics (panel B).
